# Supplementary material for: Multi‐dataset identification of innovative feature genes and molecular mechanisms in keratoconus
Source: J Cell Mol Med. 2024 Sep 19;28(18):e70079. doi: 10.1111/jcmm.70079 (PMC11412914; doi:10.1111/jcmm.70079)
Supplement: Supplementary file 2 — Material S2. [file JCMM-28-e70079-s002.zip › 7.GSEA analysis/4.MYRF/KEGG_ASTHMA.html]

Details for gene set KEGG\_ASTHMA[GSEA]

|  || Dataset | MYRF.group.cls#h\_versus\_l |
| Phenotype | group.cls#h\_versus\_l |
| Upregulated in class | l |
| GeneSet | KEGG\_ASTHMA |
| Enrichment Score (ES) | -0.6576887 |
| Normalized Enrichment Score (NES) | -1.4518752 |
| Nominal p-value | 0.07755102 |
| FDR q-value | 0.21459123 |
| FWER p-Value | 0.823 |
Table: GSEA Results Summary

  

Fig 1: Enrichment plot: KEGG\_ASTHMA      
 Profile of the Running ES Score & Positions of GeneSet Members on the Rank Ordered List

  

| SYMBOL | TITLE | RANK IN GENE LIST | RANK METRIC SCORE | RUNNING ES | CORE ENRICHMENT || 1 | IL9 | na | 3726 | 0.263 | -0.0730 | No |
| 2 | IL3 | na | 5237 | 0.215 | -0.0885 | No |
| 3 | IL13 | na | 7183 | 0.159 | -0.1238 | No |
| 4 | HLA-DOA | na | 8167 | 0.134 | -0.1345 | No |
| 5 | EPX | na | 15511 | 0.023 | -0.3432 | No |
| 6 | CCL11 | na | 15898 | 0.019 | -0.3519 | No |
| 7 | HLA-DRB5 | na | 20183 | -0.024 | -0.4722 | No |
| 8 | FCER1G | na | 22701 | -0.055 | -0.5376 | No |
| 9 | HLA-DRB3 | na | 23156 | -0.062 | -0.5425 | No |
| 10 | HLA-DQB1 | na | 24038 | -0.076 | -0.5579 | No |
| 11 | HLA-DRB1 | na | 24046 | -0.077 | -0.5481 | No |
| 12 | HLA-DOB | na | 24105 | -0.078 | -0.5397 | No |
| 13 | IL5 | na | 26211 | -0.117 | -0.5850 | No |
| 14 | HLA-DMA | na | 27498 | -0.148 | -0.6028 | No |
| 15 | IL10 | na | 28824 | -0.186 | -0.6167 | No |
| 16 | HLA-DRB4 | na | 30247 | -0.232 | -0.6274 | Yes |
| 17 | CD40LG | na | 30708 | -0.251 | -0.6080 | Yes |
| 18 | MS4A2 | na | 31419 | -0.287 | -0.5910 | Yes |
| 19 | HLA-DPA1 | na | 32053 | -0.320 | -0.5675 | Yes |
| 20 | PRG2 | na | 32084 | -0.322 | -0.5262 | Yes |
| 21 | CD40 | na | 32572 | -0.356 | -0.4939 | Yes |
| 22 | HLA-DRA | na | 33031 | -0.390 | -0.4562 | Yes |
| 23 | IL4 | na | 33045 | -0.391 | -0.4055 | Yes |
| 24 | HLA-DMB | na | 33256 | -0.414 | -0.3575 | Yes |
| 25 | FCER1A | na | 34011 | -0.524 | -0.3109 | Yes |
| 26 | HLA-DQA2 | na | 34217 | -0.576 | -0.2416 | Yes |
| 27 | TNF | na | 34328 | -0.609 | -0.1653 | Yes |
| 28 | HLA-DPB1 | na | 34331 | -0.609 | -0.0858 | Yes |
| 29 | HLA-DQA1 | na | 34602 | -0.745 | 0.0036 | Yes |
Table: GSEA details [plain text format]

  

Fig 2: KEGG\_ASTHMA      
 Blue-Pink O' Gram in the Space of the Analyzed GeneSet

  

Fig 3: KEGG\_ASTHMA: Random ES distribution      
 Gene set null distribution of ES for **KEGG\_ASTHMA**
